# Sleeping Sickness

**Published:** 1919-06

**Authors:** 


### SLEEPING SICKNESS.

An address on sleeping sickness by Dr. K. H. Beall of Fort Worth and an interesting discussion that followed the address was one of the features of the seventy-eighth and seventy-ninth semi-annual sessions of the North Texas Medical Association that convened June 3rd in Dallas for a two-days convention. The convention was the first post-war assembly of the physicians of North Texas and a considerable number of those in attendance had seen war service.

In the case discussion of sleeping sickness Dr. Beall and others who spoke recited the histories of nine cases that have developed in North Texas since the first of the year. Death resulted in three instances, but was not traceable directly to the sleeping sickness, and Dr. Beall indicated that the malady of itself is not exceedingly dangerous. Information on the subject, he said, was too meager for a formal paper and his address was entirely informal. He was surprised, he said, to find that the illness is by no means new, and when he began to search the records of the past he found that the disease had been marked as early as 1718 and has broken out at intervals of from twenty to thirty years since that date. The name sleeping sickness, he said, in unfortunate, because the malady is not at all identical with the true sleeping sickness of Africa. An epidemic of the disease that has appeared recently in this country occurred in 1890 in Italy and was given the name of nona. Dr. Beall gave other names by which the disease has been called, but favored that bestowed upon it by Italy.

### Appeared in Australia Last Year.

According to Dr. Beall's recital the present epidemic appeared in Australia early last year and was reported by an Australian medical observer. It broke out a little later in England and a British commission was formed to conduct an investigation. The disease first appeared in the United States early last winter.

The etiology of the disease is little known, Dr. Beall said, but there seems to be plenty of evidence to establish the fact that it has followed in the wake of other outbreaks of influenza, and that it surely has followed influenza in this country. There is a difference of opinion as to whether afflicted persons have first suffered attacks of influenza.

The most common symptoms pointed out by Dr. Beall are: Slight fever at first, followed by slowly developing lethargic state, nasal twang to voice, abnormality of spine fluid, face void of expression as if paralyzed. In almost every instance the sufferer has the appearance of being asleep, but almost never is, Dr. Beall said, and not infrequently insomnia goes as a part of the disease. Usually the recovery comes as gradually as the disease develops.

---

### Two Interesting Cases.

Editor Texas Medical Journal,
Austin, Texas.

Replying to your request of the 14th inst. in which you ask a report of the case of Sleeping-sickness that I reported to Dr. Garison, I will say I have treated two cases and will report each of them.

(1) I was called March 14th, 1919, to see Mrs. R. J. T.:—Found her with swollen tongue covered with a tongue slime or mucus, peristalic action of the upper bowels practically nil, liver swollen and engorged, complained of a suffering that she said was not pain but a sort of restless sensation as if she was about to have cramping of all her muscles, had the influenza about Christmas and had not been well since, but appetite strong, aged 79.

My first diagnosis was Intestinal indigestion or Mal-assimilation; immediately gave her, for first dose 2 C. C. pills, with instructions to repeat one an hour till a good action from bowels and liver was produced. In about two hours I was called back with a message stating she was much worse. When I arrived I found she was hysterical, but otherwise the same as on my first visit. For the Hysteria I gave her for the first dose 2 pellets each composed of Morphia Sul. 1/16 gr., Calomel 1/8, Capsicum 1/16, Pwd. Ipicac 1/32, and Pwd. Camphor 1/16 grs., with instructions to repeat one an hour till relieved—but one more dose was needed (3 pellets) and when quiet she went to sleep and never fully awoke again, living in that sleeping stupor eight full days, dying on the ninth day of her last illness. Nine of the C. C. pills were given before an action on the bowels was effected and then by aid of an enema—the liver never acted.

(2) J. H. F.—on March 14, came to my office for a bottle of Foley's Kidney Pills, saying his kidneys acted too much and with a scalding sensation; the 26th he came again to my office, saying: That he had trouble that morning laying off corn ground, that when he looked at one row he saw two, and besides he was so sleepy that he found it a difficulty to keep awake while at work—he asked me to give him something that would keep him from seeing more than he was looking at, and to keep him awake. I examined him and told him that there was no specific medicine nor medicines that would of themselves perform such wonders, but that his blood was poisoned with the "Flu" that he had had the first of the previous January, that his liver was lazy and his assimilation bad, that I could give him something that would help nature to cleanse the foul blood and encourage the liver to proper action and that at present is all that the medical science has advised to be done for such cases. His tongue, liver and bowels were in identically the same condition as those of case No. (1), but not so intense. He was aged 74, 5:10 tall, weight about 200 lbs. Treatment: Liver medicine (C. C. pills) 6 to 12 daily—was needed to produce very modest actions on the bowels and liver. The first week I visited him daily and usually found him asleep, and if not asleep when I arrived he would go to sleep while I was there, in spite of the most interesting entertainment we could give him. We have kept the liver medicine up regularly and each week we could see his condition was surely but slowly approaching the normal till now his subnormal strength is all that we can see of him that is not normal.

In view of the fact that the symptoms in the two cases were similar, to-wit: Each had the "Flu" recently, that the physical symptoms in each case were alike, and each the same essentially that they were when they had the "Flu," I am of the opinion that, if there ever is a specific germ found to cause the disease, it will be found in the mucus on the tongue of the patients, and in the alimentary tract especially in the upper bowel, that it will be found synthetical in nature, that is to say—produced as a result of the "Flu" germs that the system has not yet eliminated and a resultant impure bile.

The best diet I believe in all these cases should be limited to a reasonable quantity, as is often difficult to do on account of the gormandizing appetite present with the mild cases which are the only hopeful cases of this strange disease. Raw eggs and sweet milk well trituated together given moderately every three hours with light bread toast.

Yours very truly,

G. A. E. MARTIN, M. D.,
Adono, Arkansas.

---

### Treatment and Recovery.

Commanche, Okla., June 10, 1919.

Mrs. B., age 40, married, seven children all living, always has been strong and healthy; had a light attack of influenza in January, 1919, with no pneumonia following, and apparent full recovery.

In February, about a month afterward, I found her with a temperature running from 99½ to 101, which lasted three or four days. There were no aches, no pains, no nausea, but feeling very weak and exhausted; heart and lungs negative. Slight albuminuria, no indican, no acetone, no casts, no pus cells, sp. gr. 1020. Nothing abnormal in the differential blood count. I was so busy that I did not make a total count. I have forgotten the blood pressure, but think it was about 100. The temperature lasted three or four days and quit, when she began to have an increasing drowsiness and complained of "seeing double."

The drowsiness was worse in the afternoons; could not stay awake, would go to sleep while being talked to; awakened, only to drop off to sleep again in a few seconds. Could always be awakened, and sometimes would get up, if permitted, to lie down and go to sleep again. In about two weeks the drowsiness began to disappear, until in about eight weeks from the beginning of the attack had about all disappeared. But even yet, June 10, she tells me that she still feels sluggish, easily exhausted, not fairly on her feet. The diplopia behaved about the same way and now is entirely gone, but eyes tire easily.

I have not seen her, now, professionally for some weeks, but she is still easily exhausted and complains of some "heart trouble;" just what it is I have had no opportunity to find out. Possibly some infection.

In the treatment she took very little medicine. Free purgation at the beginning, eliminants, chiefly sodium citrate, *rest in bed*, and proper feeding.

For some weeks now she has been able to attend to her household duties.

C. M. HARRISON, M. D.

---

**A Case of Encephalitis Lethargica.**

Wm. B., colored, whom I had treated several times during the course of five years for swamp fever presented himself to me during March, 1919, with a disease that showed a number of symptoms of the so-called ''sleeping sickness.''

He had been under the treatment of another doctor who seems to have accepted the patient's own diagnosis that his condition had been due to the limbs and trunks of trees falling and striking him on the head in the pursuit of his work as a timberman. But as there was no sign of fracture or even of concussion of the brain in the history of the patient's mishaps as a logger, I could not accept this diagnosis.

The patient presented a picture of distress and a great deal of confusion unless forcing himself to concentrate his mind. The patient complained of dizziness to the point of pain (not pain in the head to the point of dizziness). His pupils reacted sluggishly; he complained of distress in looking at anything for any length of time ending in an overpowering wish to sleep; in fact, the desire to sleep was constantly with him. Between this and his feeling of weakness to the point of faintness, his only relief was sleeping.

All reflexes were dull, the patellar reflexes almost absent. The patient could not stand without falling in the erect position with eyes closed and arms extended. Pulse was always about 110 and temperature 100 F.

The greatest complaint was about the intense dizziness and the extreme weakness.

I put him on extract of adrenal gland and of the spermatic secretion which greatly strengthened him. He still complained of dizziness and I had him shaved on the scalp and rubbed inunctions of mercury and iodine separately and at separate times. I administered mercurials and indeed treated him as a syphilitic. (There was no history of syphilis). The patient although patently better to me claimed he was not better and dropped all medicine after 4 weeks treatment and went away. I saw him again two weeks later and put him back on the treatment against his protests; his wife sided with me. The second reaction to the medical treatment was marvelous and he improved so markedly that he admitted it himself. He is now back at his old job.

DR. G. C. BOUDOUSQUIE,
1520 Polymnia Street,
New Orleans, La.

---

**Symptoms of Recovery.**

Jeannette, La., June 16, 1919.

Texas Medical Journal:

Your request of several days ago regarding a paper on my case of sleeping sickness came, and was laid aside for reply.

Well, my case showed up about this way:

A girl, 10, thin and emaciated generally,not particularly from flu which she had had about six weeks previous, but from poor and insufficient food or money so common in this part of the country.

She became ill by getting weaker, with a headache and severe pain in her back. I was called six days after she was in bed unable to go any longer. I found she had been lounging around the house with a few clothes on and it was mid-winter.

When seen by me they had put her to bed with all her clothes on, so I had her changed up and put to bed right, before I proceeded with my examination. The first thing I noticed was the complete lethargy and the way she lay in bed—a perfect cadaver to all appearances, eyes closed and when forced to open the balls diverged and converged as well as rolled every way. This aroused my suspicion as to the possibility of having a case of lethargy encephalitis so I went over the spine. Neck was rigid but straight, limbs also were slightly stiff, especially affected. The spine from the dorsal region was stiff. The child could be raised without bending spine. Arms and legs fell as though dead but on pinching or sticking the skin quite severely the child would groan and move limbs fairly well.

The temperature ran from 99½ to 101, never over. The jaw was freely movable and the child could be aroused after considerable manipulating of the face and shaking and would then drink a small amount of milk or soup and broth, and immediately lapse back into a state of quiet and sleep that had the appearance of moderate coma which would last until she aroused or she felt a desire to move bowels or urinate, but not every time, for frequently she would urinate in the bed unconsciously.

This drowsiness lasted about 22 or 23 days as best I could figure and then she began to recover with a fall of temperature to 95½ which gradually came to 98 on forced feeding and in five weeks she was back to about where she started and is now very well indeed except for the loss of a few reflexes and prolysis of a muscle here and there in the legs and arms. This seems not to interfere with her general movements, or speech or eyesight.

The case was seen by Dr. Jones, U. S. Army Health Department, after recovery.

Hoping this will reach you and be open to criticism, as it is as I saw it, and not from the journals.

I am yours very truly,

P. A. BOYKIN, M. D.

---

### NEW SUBSCRIPTIONS.

The Texas Medical Journal acknowledges with thanks the following new subscriptions this month, all of which have paid a year in advance:

Dr. C. C. Cate, Morgan, Texas; Dr. J. T. Seale, Neches, Texas; Dr. A. B. Jones, Box 114, Richmond, Texas; Dr. B. F. Crabtree, Midlothian, Texas; Dr. J. I. Pearson, Joshua, Texas; Dr. W. L. Hester, Brownwood, Texas; Dr. Wm. P. Coyle, Orange, Texas; Dr. R. S. Sutton, Box 32, Bartlett, Texas; Dr. R. D. Williamson, Inez, Texas; Dr. H. Reid Robinson, 704 American Natl. Ins. Bldg., Galveston, Texas; Dr. G. D. Small, Box 69, Palestine, Texas; Dr. T. C. McCloud, Box 81, Jermyn, Texas; Dr. C. M. Wright, Rock Island, Texas; Dr. W. M. Jenkins, Italy, Texas; Dr. W. S. Tyson, New Boston, Texas; Dr. Jas. A. Caldwell, McKinney, Texas; Dr. E. H. Scarborough, Brushy Creek, Texas; Dr. W. L. Gaines, Bomarton, Texas; Dr. J. W. Smith, La Grange, Texas; Dr. C. L. Root, Colorado, Texas; Dr. A. T. Reed, Girard, Texas; Dr. R. L. Hamilton, Matador, Texas; Dr. J. W. Decker, 2520 State St., Dallas, Texas; Dr. E. A. Lee, Gordon City R. F. D., Big Springs, Texas; Dr. O. F. Strong, Box 208, Gainesville, Texas; Dr. W. E. Ramsey, 618 Union National Bank Bldg., Houston, Texas; Dr. P. L. Smith, 2008 N. Main St., Houston, Texas; Dr. W. A. Garrett, 1417 Washington St., Houston, Texas; Dr. T. B. Busbee, Box 176, Rising Star, Texas; Dr. E. L. Byrd, Clint, Texas; Dr. R. C. Shanks, Bells, Texas; Dr. J. T. Jones, Fluvanna, Texas; Dr. W. T. White, Wilson Bldg., Dallas, Texas; Dr. E. G. Magruder, San Angelo, Texas; Dr. C. F. Winfield, Rye, Texas; Dr. J. L. Austin, Rockwall, Texas; Dr. Edward White, 926 Maddox Ave., Ft. Worth, Texas; Dr. J. M. McCuan, Farwell, Texas; Dr. D. W. Black, Lampasas, Texas; Dr. L. E. Petty, Forestburg, Texas.

